# Ultra-high pressure balloon angioplasty for pulmonary artery stenosis in children with congenital heart defects: Short- to mid-term follow-up results from a retrospective cohort in a single tertiary center

**DOI:** 10.3389/fcvm.2022.1078172

**Published:** 2023-01-23

**Authors:** Shuliang Xia, Jianbin Li, Li Ma, Yanqin Cui, Techang Liu, Zhouping Wang, Fengxiang Li, Xumei Liu, Shan Li, Lu Sun, Lin Hu, Yubin Liu, Xun Ma, Xinxin Chen, Xu Zhang

**Affiliations:** ^1^Department of Cardiovascular Surgery, Guangzhou Women and Children's Medical Center, Guangzhou Medical University, Guangzhou, China; ^2^Guangdong Provincial Key Laboratory of Research in Structural Birth Defect Disease, Guangdong Provincial Clinical Research Center for Child Health, Guangzhou, China; ^3^Department of Echocardiogram Room, Guangzhou Women and Children's Medical Center, Guangzhou Medical University, Guangzhou, China; ^4^Department of Pediatric Cardiology, Guangzhou Women and Children's Medical Center, Guangzhou Medical University, Guangzhou, China; ^5^Department of Interventional and Vascular Anomalies, Guangzhou Women and Children's Medical Center, Guangdong Provincial Clinical Research Center for Child Health, Guangzhou Medical University, Guangzhou, China

**Keywords:** pulmonary artery stenosis, ultra-high pressure balloon angioplasty, primary treatment, infants, children

## Abstract

**Objective:**

Balloon angioplasty (BA) has been the treatment of choice for pulmonary artery stenosis (PAS) in children. There remains, however, a significant proportion of resistant lesions. The ultra-high pressure (UHP) balloons might be effective in a subset of these lesions. In this study, we analyzed the safety and efficacy with short- to mid-term follow-up results of UHP BA for PAS in children with congenital heart defects (CHD) in our center.

**Methods:**

This is a retrospective cohort study in a single tertiary heart center. Children diagnosed with PAS associated with CHD were referred for UHP BA. All data with these children were collected for analysis with updated follow-up.

**Results:**

A total of 37 UHP BAs were performed consecutively in 28 children. The success rate was 78.4%. A significantly (*P* = 0.005) larger ratio of the balloon to the minimal luminal diameter at the stenotic waist (balloon/waist ratio) was present in the success group (median 3.00, 1.64–8.33) compared to that in the failure group (median 1.94, 1.41 ± 4.00). Stepwise logistic regression analysis further identified that the balloon/waist ratio and the presence of therapeutic tears were two independent predictors of procedural success. The receiver operating characteristic curve revealed a cut-off value of 2.57 for the balloon/waist ratio to best differentiate success from failure cases. Signs of therapeutic tears were present in eight cases, all of whom were in the success group. Perioperative acute adverse events were recorded in 16 patients, including 11 pulmonary artery injuries, three pulmonary hemorrhages, and two pulmonary artery aneurysms. During a median follow-up period of 10.4 (0.1–21.0) months, nine cases experienced restenosis at a median time of 40 (4–325) days after angioplasty.

**Conclusions:**

The UHP BA is safe and effective for the primary treatment of PAS in infants and children with CHD. The success rate is high with a low incidence of severe complications. The predictors of success are a larger balloon/waist ratio and the presence of therapeutic tears. The occurrence of restenosis during follow-up, however, remains a problem. A larger number of cases and longer periods of follow-up are needed for further study.

## 1. Introduction

Pulmonary artery stenosis (PAS) is defined as a narrowing of any part of the pulmonary artery tree. It may be located in the main pulmonary artery trunk, adjacent to the bifurcation, or involved unilaterally or bilaterally at the ostial, lobar, segmental, or subsegmental areas. Most cases of PAS become complicated by congenital heart defects. Patients with pulmonary atresia (PA), ventricular septal defect (VSD), and tetralogy of Fallot (ToF) have been reported to have a 70, 20, and 10% chance, respectively, of developing branch PAS throughout the natural history of the disease (1, 2). PAS increases right ventricular pressure, leading to ventricular hypertrophy and restriction of filling, which eventually progresses to heart failure or malignant arrhythmias. Cyanosis, hypoxemia, and metabolic acidosis may also occur if the patient has a ventricular or atrial shunt. Unilateral PAS results in a perfusion imbalance in the bilateral lung fields. The pulmonary vascular bed on the affected side might be dysplastic due to inadequate perfusion, while the contralateral side develops congestion and recurrent infections.

Traditional surgical correction is more invasive and has a higher complication rate, which has made balloon angioplasty (BA) the cornerstone of current PAS treatment since its introduction in the 1980's (1, 3, 4). However, there remains a significant proportion of lesions that do not respond to simple BA (2). Cutting BA (5–11) and endovascular stenting (12–18) offer promising options for these lesions. Cutting BA is effective in increasing lumen diameter, especially in lesions located distally (6, 9, 11). However, the relatively high restenosis rate (9, 11, 19) limits its widespread use. Percutaneous transluminal pulmonary artery stenting is a technically more successful procedure (3, 16, 17, 20–22) with sustained hemodynamic improvement (23) and low restenosis rates (24), as well as fewer procedural adverse events (21, 24–27). However, the ongoing demand for physical development in infants and young children limits the application of stenting in this group, which constitutes the vast majority of the PAS population.

Ultra-high pressure (UHP) balloon catheters were initially used for the treatment of stenotic hemodialysis fistulas (28). The UHP balloon catheters are layered with cross-matrix woven ultrahigh molecular weight polyethylene, which potentiates them with rated pressures up to 30 atm. They can, in reality, allow a pressure higher than 30 atm without rupture. In recent years, *in vitro* and animal experiments (29, 30), as well as preliminary studies (31, 32), with UHP BA have been carefully explored in specialized conditions. The aim of these studies has almost always been to re-expand previously implanted smaller scaffolds to match the somatic growth of infants and young children. Until recently, there has been a lack of experience with initial UHP BA for PAS lesions. Does the UHP balloon further improve the outcome of simple BA? Are UHP balloons as safe as conventional balloons? What are the major adverse events for UHP BA and their respective incidence? Could UHP BA be the treatment of choice for infants and young children with PAS? In this study, we summarized the outcomes of UHP BA for the primary intervention of PAS in our center in an attempt to gain some experience for the questions above mentioned.

## 2. Materials and methods

### 2.1. Patients' inclusion

Children diagnosed with PAS associated with CHD were screened from May 2020 to September 2021 at Guangzhou Women and Children's Medical Center. The indications for BA were in line with universally accepted criteria (1, 3, 24, 33) with slight modifications. Specifically, patients who met one of the following criteria were enrolled for UHP BA: (1) patients who were symptomatic; (2) patients with the pressure gradient across the stenotic segment ≥20 mm Hg; and (3) patients with either the right ventricular systolic pressure ≥60 mm Hg or the ratio of right to left ventricular systolic pressure ≥0.5. Imbalanced bilateral lung perfusion for assessment of unilateral PAS was not adopted as one of the criteria because radionuclide lung perfusion imaging was not available in our center.

### 2.2. Procedures and follow-up

Routine left and right heart catheterizations were performed in all patients for hemodynamic assessments. Right ventriculography or main pulmonary arteriography was performed to demonstrate the detailed anatomy at the stenotic sites. The Conquest^®^ (nominal pressure 8 atm, rated burst pressure 20–30 atm) and Atlas^®^ (nominal pressure 4–6 atm, rated burst pressure 12–18 atm) PTA balloon dilatation catheters (Bard Peripheral Vascular, Inc., U.S.A.) were used for UHP BA with a basixCOMPAK™ inflation device (Merit Medical Systems, Inc., U.S.A.). The selected balloon size was determined by a combination of several factors: (1) the initial balloon-to-waist ratio was chosen as 2–3, with a serial increase to 3–5 until success was achieved, or the risk of complications was considered too high to further increase the balloon size; (2) the maximal balloon diameter should not exceed two times the normal diameter of the adjacent vessel to the lesion; and (3) the final diameter of the dilated site recoiled to 60–70% of the balloon diameter after angioplasty, which could be used to predict the appropriate bilateral lung perfusion.

During dilation, the nominal pressure of the selected balloon was first achieved. If the waist of the balloon still existed, the pressure was increased to eliminate the waist until it reached the rated burst pressure. If the waist still existed, the pressure was further slowly increased with caution to a maximum of 30 atm. Once the waist disappeared, inflation was immediately terminated, and the pressure was maintained for up to 30 s until the patient suffered from desaturation. We did not encounter balloon rupture in this group of interventions.

### 2.3. Post-angioplasty monitoring and follow-up

After dilation, patients were closely monitored in the cardiac intensive care unit (CICU) in our center. The mean (9.4 ± 10.9) hours of mechanical ventilation was performed in an attempt to avoid post-angioplasty adverse events such as pulmonary edema or hemorrhage. After the gradual withdrawal of ventilatory support, patients were discharged and regularly followed at the outpatient clinic. Serial electrocardiograms and transthoracic echocardiograms were essential during follow-up. CT scan or pulmonary arteriography was performed at least 3 months after angioplasty and repeated during follow-up when necessary.

### 2.4. Criteria for procedural success and restenosis

The well-recognized criteria for procedural success and restenosis (1–3, 24, 33–38) were adopted in our study with slight modifications. The procedure was considered a success if one of the following criteria was met:

(1) A ≥ 50% increase in the vessel diameter at the stenotic site;
(2) A ≥ 50% decrease in the pressure gradient across the stenosis;
(3) A ≥ 20% decrease in the ratio of right to left ventricular systolic pressure or the ratio decreased to below 0.5.

Restenosis was defined as a ≥50% decrease in the initial gain of the vessel diameter after angioplasty during follow-up.

### 2.5. Data collection and analyses

Patient demographics, clinical data, electrocardiograms, echocardiograms, chest roentgenograms, computed tomography scans, as well as other available data were collected along with hemodynamic and angiographic parameters obtained from the catheterization lab. Complications during and immediately after the angioplasty, as well as follow-up outcomes, were also collected.

Normality tests were performed for all continuous variables. Univariate comparisons were performed either by the two-tailed student *t*-test for variables that were normally distributed or by non-parametric tests for those not normally distributed. Variables that were significantly different between the success group and the failure group were analyzed by stepwise logistic regression. The rates between the two groups were compared by the chi-square test. The receiver operating characteristic curve was depicted to evaluate the ability of the variable to differentiate both groups, and a cut-off value was determined for best distinction. A *P*-value of < 0.05 was considered significant.

The study was conducted according to a protocol approved by the Ethics Committee for Clinical Investigations at our center. All the authors had full access to the data and take responsibility for its integrity. All the authors had read and agreed to the manuscript as written.

## 3. Results

### 3.1. Study patients

Twenty-eight patients were included in this study, with male predominance (20/28). The baseline patient demographics are provided in Table 1. The primary diagnoses were mainly PA/VSD and ToF (22/28). The average age of this group at angioplasty was 62.8 ± 37.6 months, with an average weight of 16.8 ± 8.7 kg and an average height of 103.2 ± 19.2 cm. The preoperative percutaneous oxygen saturation was 92.3 ± 7.6%. The NYHA functional classes were all I or II (two patients were in class I and 26 patients were in class II). The cardiothoracic ratio on chest roentgenogram was increased in 89% of patients (25/28), with a mean ratio of 0.61 ± 0.06. The preoperative electrocardiograms were mainly right bundle branch block (RBBB) or right ventricular high voltage (20/28), as illustrated in Table 2. All patients demonstrated right ventricular hypertrophy or right ventricular enlargement (except for the single ventricle case) on echocardiograms, with varying degrees of tricuspid regurgitation (TR) and pulmonary regurgitation (PR). The mean preoperative TR area was 4.6 ± 3.0 cm^2^/m^2^ body surface area. Many PRs were severe or moderate since this portion of patients experienced a right ventricular outflow tract reconstruction with a valved conduit made of a bovine jugular vein. This xenograft had a trend of decay years after surgery. The preoperative valve regurgitations are summarized in Table 3.

**Table 1:** Primary diagnoses of patients.

**Primary diagnoses**	**No. patients**	**Percentage, %**
PA/VSD	16	57
ToF	6	21
UAPA	2	7.1
DORV	1	3.6
SV after Fontan	1	3.6
Congenital left PAS/RAOA/ALSCA	1	3.6
CAT	1	3.6

PA, pulmonary atresia; VSD, ventricular septal defect; ToF, Tetralogy of Fallot; DORV, double outlet right ventricle; UAPA, unilateral absence of pulmonary artery; SV, single ventricle; PAS, pulmonary artery stenosis; RAOA, right aortic arch; ALSCA, aberrant left subclavian artery; CAT, common arterial trunk.

**Table 2:** Preoperative electrocardiograms of patients.

**Electrocardiographic diagnoses**	**No. patients**	**Percentage, %**
RBBB	18	67
With RVE or RVHV	8	
With prolonged PR interval	1	
With sinus bradycardia	1	
RVHV	3	11
IVB	2	7.1
LVE or LVHV	2	7.1
LBBB	1	3.6
Normal	1	3.6
N/A	1	3.6

RBBB, right bundle branch block; RVE, right ventricular enlargement; RVHV, right ventricular high voltage; IVB, intraventricular block; LVE, left ventricular enlargement; LVHV, left ventricular high voltage; LBBB, left bundle branch block; N/A, not applicable.

**Table 3:** Preoperative valve regurgitation on echocardiograms of patients.

**Degrees of valve regurgitation**	**No. patients**	**Percentage, %**
**Tricuspid regurgitation**		
Severe	1	3.6
Moderate	8	29
Mild	12	43
Trivial	6	21
None	1	3.6
**Pulmonary regurgitation**		
Severe	12	43
Moderate	5	18
Mild	1	3.6
Trivial	2	7.1
None	8	29

### 3.2. Immediate effects of angioplasty

A total of 37 UHP balloon angioplasties were performed in 28 patients, including 21 procedures on the left pulmonary artery, 13 on the right pulmonary artery, two on the main pulmonary artery, and one on the upper branch of the right pulmonary artery. A total of 37 UHP balloons were utilized, including 32 Conquest^®^ balloons (four of 5 mm size, five of 6 mm size, nine of 8 mm size, five of 10 mm size, and nine of 12 mm size) and five Atlas^®^ balloons (three of 14 mm size, and two of 16 mm size), with a ratio of the balloon to the minimal luminal diameter at the stenotic waist (balloon/waist ratio) of 3.6 ± 1.9. During the procedures, three pre-dilations were performed due to extreme stenosis, with one PTA balloon and two PTCA balloons. The average procedural time was 107.8 ± 44.6 min, with a fluorescence exposure time of 31.3 ± 26.8 min and a cumulative radiation dose of 100.0 ± 116.5 mGy. The average hospitalization cost was RMB ¥50,320 ± 14,351, which equaled USD $7,455 ± 2,126 at the time of the research.

The increase of the stenotic diameter was 2.9 ± 2.4 mm immediately after angioplasty, which represented an improvement of 97.3 ± 86.0%. The pressure gradient across the stenosis decreased from 32.0 ± 16.0 mm Hg to 15.3 ± 17.4 mm Hg, which represented an improvement of 54.2 ± 38.0%. The ratio of right to left ventricular systolic pressure decreased from 0.83 ± 0.23 to 0.72 ± 0.26, which represented an improvement of 12.2 ± 21.7%. The detailed parameters are provided in Table 4.

**Table 4:** Angioplastic parameters during procedure.

**Items**	**Pre-dilation**	**Post-dilation**	***P*-value**
Diameter of stenosis (mm)	3.8 ± 2.0	6.7 ± 3.4	0.000
Indexed by BSA (mm/m^2^)	6.7 ± 4.6	11.8 ± 8.1	0.000
Increase in diameter of stenosis (%)	97.3 ± 86.0	
PG across stenosis (mmHg)	32.0 ± 16.0	15.3 ± 17.4	0.000
Indexed by BSA (mmHg/m^2^)	56.5 ± 35.4	25.0 ± 25.8	0.000
Decrease in PG across stenosis (%)	54.2 ± 38.0	
RVSP/LVSP (or AOSP)	0.83 ± 0.23	0.72 ± 0.26	0.004
Decrease in RVSP/LVSP (or AOSP; %)	12.2 ± 21.7	
Balloon/waist ratio	3.6 ± 1.9	

PG, pressure gradient; RVSP, right ventricular systolic pressure; LVSP, left ventricular systolic pressure; AOSP, aortic systolic pressure; Balloon/waist ratio, the ratio of balloon to the minimal luminal diameter at stenotic waist before dilation.

### 3.3. Angioplasty success and its predictors

**Figure 1:**
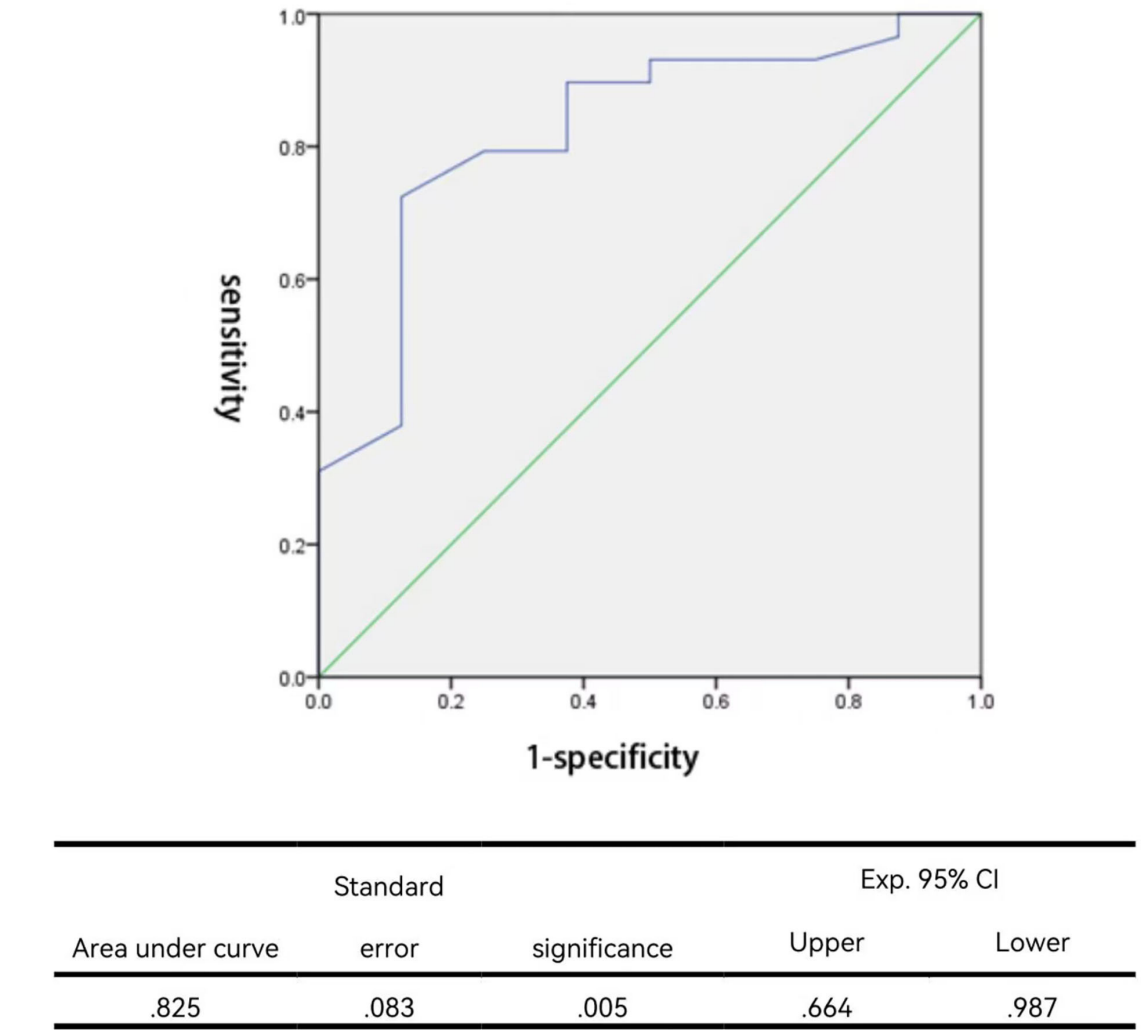
Receiver operating characteristic curve for the balloon/waist ratio to predict angioplastic success.

According to the previously described criteria, angioplasties achieved success in 29 procedures and failed in eight procedures. The success rate was 78.4%. For those who succeeded, the median balloon/waist ratio was 3.00 (1.64–8.33) in the success group, while the median ratio was 1.94 (1.41–4.00) in the failure group. A significantly larger balloon/waist ratio was present in the success group (*P* = 0.005). Stepwise logistic regression analysis further identified that the balloon/waist ratio and the presence of therapeutic tears were two independent factors to predict procedural success (Table 5). The receiver operating characteristic curve was drawn to evaluate the ability of the balloon/waist ratio to predict angioplasty success, with the area under the curve of 0.825 (Figure 1). A cutoff value of 2.57 was selected for the best differentiation of the success group from the failure group, with a sensitivity of 0.724 and a specificity of 0.875 (Table 6).

**Table 5:** Stepwise logistic regression analyses for predictors of procedural success.

**Items**	**Success group**	**Failure group**	*P*-value^*^	Significance^**^
	**(*n* = 29)**	**(*n* = 8)**		
Male gender (No./percentage)	19/65.5%	7/87.5%	0.240	0.712
Age at angioplasty (months)	63.8 ± 37.1	59.6 ± 41.9	0.785	0.456
Body weight (kg)	16.9 ± 9.3	16.4 ± 6.0	0.889	0.906
Height (cm)	102.3 ± 19.3	106.5 ± 19.7	0.593	0.842
Diameter of stenosis (mm)	3.5 ± 1.8	4.9 ± 2.3	0.085	0.415
Diameter of balloon (mm)	10.8 ± 2.8	9.6 ± 2.7	0.279	0.052
**Balloon/waist ratio**	**3.9** **±2.0**	**2.2** **±0.8**	**0.029**	**0.05**
Increase/stenosis ratio	1.1 ± 0.9	0.3 ± 0.1	0.017	0.997
Balloon/vessel ratio	1.9 ± 0.7	1.6 ± 0.6	0.431	0.649
**Presence of therapeutic tear** **(No./percentage)**	**8/27.6%**	**0/0%**	**0.16**	**0.001**

Balloon/waist ratio, the ratio of balloon to the minimal luminal diameter at stenotic waist; Increase/stenosis ratio, the ratio of the post-angioplastic increased diameter to original diameter at the stenotic site; Balloon/vessel ratio, the ratio of the balloon diameter to the vessel diameter at the stenotic site after angioplasty.

* From two-tailed student t-test or Chi square test.

** From stepwise logistic regression analysis. The bold values indicate the statistically significant.

**Table 6:** Determination of the cut-off value for the balloon/waist ratio.

**Cut-off value**	**Sensitivity**	**Specificity**	**Youden index**
0.4118	1	0	0
1.5256	1	0.125	0.125
1.6768	0.966	0.125	0.091
1.746	0.931	0.25	0.181
1.8264	0.931	0.375	0.306
1.9179	0.931	0.5	0.431
1.9804	0.897	0.5	0.397
2.1364	0.897	0.625	0.522
2.2834	0.862	0.625	0.487
2.3137	0.828	0.625	0.453
2.3567	0.793	0.625	0.418
2.44	0.793	0.75	0.543
**2.5658**	**0.724**	**0.875**	**0.599**
2.6365	0.69	0.875	0.565
2.7161	0.621	0.875	0.496
2.8239	0.586	0.875	0.461
2.8831	0.552	0.875	0.427
2.9545	0.517	0.875	0.392
3.0789	0.483	0.875	0.358
3.2811	0.448	0.875	0.323
3.4263	0.414	0.875	0.289
3.7241	0.379	0.875	0.254
4.069	0.31	1	0.31
4.4219	0.276	1	0.276
4.8529	0.241	1	0.241
5.1667	0.207	1	0.207
5.6078	0.172	1	0.172
6.5775	0.138	1	0.138
7.6364	0.103	1	0.103
8.1667	0.069	1	0.069
9.3333	0	1	0

The bold ratio indicates the cut-off value of 2.5658, which can most accurately differentiate the success group from the failure group, and the Youden index was the largest for this cut-off value.

Signs of vessel tear (also termed “therapeutic tear”) were visible in eight post-angioplasty angiograms, including intimal irregularities, vessel extravasations, and non-obstructive intra-luminal filling defects (intimal flaps) at the dilation site. All eight therapeutic tears were in the success group. None of those who failed the procedure had signs of therapeutic tears. Therefore, the presence of therapeutic tears was predictive of technical success, with a specificity of 100%, a sensitivity of 27.6%, a positive predictive value of 100%, and a negative predictive value of 27.6% (Table 7).

**Table 7:** Predictive value of the signs of “therapeutic tears”.

**Signs of therapeutic tears**	**Angioplastic success**	**Angioplastic failure**
Presence	8	0
Absence	21	8

Sensitivity = 0.276.

Specificity = 1.000.

Positive predictive value = 1.000.

Negative predictive value = 0.276.

### 3.4. Procedural adverse events

**Figure 2:**
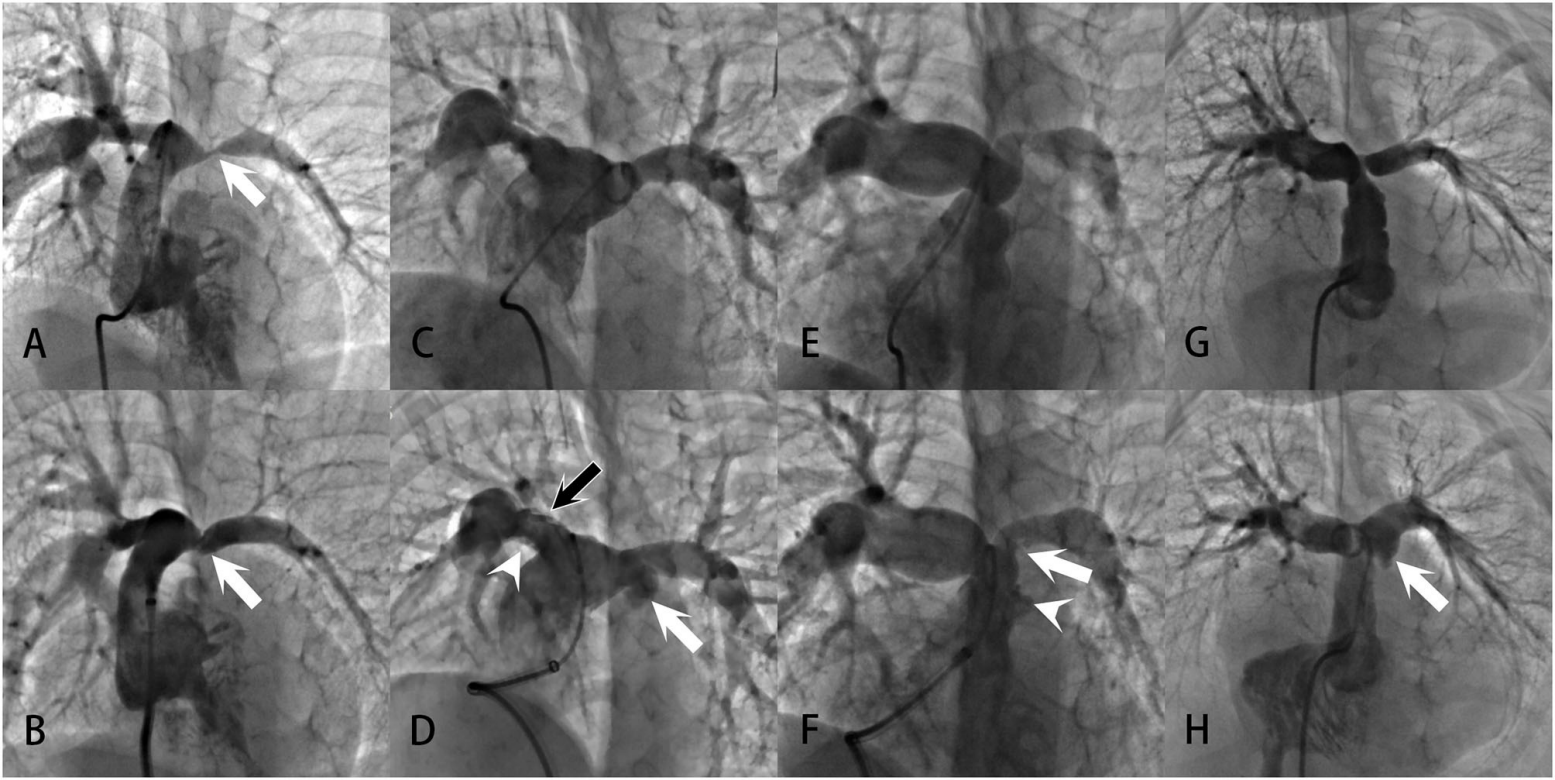
Examples of pulmonary artery injuries and formation of pulmonary artery aneurysms demonstrated in the angiograms: **(A, B)** Severe left PAS in a patient with repaired UAPA. After successful angioplasty, the vessel showed intimal irregularities (white arrow), a sign of vessel tear at the intima and media. **(C, D)** Bilateral branch PASs in a patient with repaired PA/VSD. After angioplasty, intimal irregularities (white arrowhead) and multiple filling defects (black arrow) were present at the upper and lower edge of the right pulmonary artery. Swinging of the filling defects with the cardiac cycle could be seen in the vessel, suggesting the formation of intimal flaps. Vessel extravasation occurred at the left pulmonary artery (white arrow). These were all signs of vessel tears. And the extravasation was large enough to meet the criteria of a pulmonary artery aneurysm. **(E, F)** Prominent PASs at the main and left pulmonary arteries in a patient with repaired ToF. After angioplasty, there were vessel intimal irregularities and extravasation at the main pulmonary artery (white arrowhead), indicative of vessel tear. An intimal flap was noted at the left pulmonary artery (white arrow). **(G, H)** Left PAS in a patient with repaired PA/VSD. After angioplasty, localized vessel extravasation and aneurysm formation was noted at the left pulmonary artery (white arrow).

No perioperative death occurred. During the procedures, no hemodynamic instability was recorded in any patient. Perioperative acute adverse events were recorded in 16 patients, with 11 cases of pulmonary artery injuries, three cases of pulmonary hemorrhage, and two cases of pulmonary artery aneurysm. Pulmonary artery injuries occurred after nearly 30% of the total angioplasties. Most of them, nevertheless, were signs of therapeutic tears, such as pulmonary artery vessel tears, vessel extravasations, and vessel intimal flaps (Figure 2). At the dilation site, various degrees of localized dilation were present. It was not considered an aneurysmal formation until the luminal diameter exceeded two times the adjacent normal vessel (Figure 2) and the two aneurysms showed no signs of rupture. All three pulmonary hemorrhage cases were treated with conservative strategies, including mechanical ventilation with positive end-expiratory pressure and hemorrhages resolved within 53 h. Neither pulmonary edema nor any new arrhythmias were noticed in this series. No emerging or aggravated tricuspid and pulmonary regurgitations were seen after the procedures.

### 3.5. Follow-up

The median period of follow-up was 10.4 months, ranging from 0.1 to 21.0 months. No death occurred during follow-up. At the last follow-up, the average weight was 28.6 ± 9.2 kg, with an averaged cardiothoracic ratio of 0.62 ± 0.05. During follow-up, nine cases experienced restenosis, making a restenosis rate of 24.3%. Restenosis occurred at a median follow-up time of 40 (4–325) days after angioplasty. For the nine restenosis cases, eight occurred in the group with a balloon/waist ratio >2.57. Only one restenosis occurred in the group with a balloon/waist ratio of < 2.57, which is significantly less than the larger balloon group (*P* = 0.047). This unusual result indicated that a larger balloon/waist ratio was correlated with a greater risk of restenosis.

The two pulmonary artery aneurysms did not progress during the whole follow-up. No extra pulmonary hemorrhage occurred during the follow-up. Two cases of the follow-up ECG progressed from normal to RBBB and one case recovered from RBBB to normal. The other case had an unaltered ECG during follow-up. The tricuspid and pulmonary regurgitations were not significantly altered at the last follow-up compared to the time points of pre-angioplasty, immediate post-angioplasty, and discharge, as was demonstrated by serial transthoracic echocardiography.

## 4. Discussion

Contemporary treatment strategies for PAS include simple BA, cutting BA, and endovascular stenting. Prior to the introduction of BA (1, 3, 4), surgical correction was the only option with variable but unsatisfactory results (39). In the early 1980's, Lock et al. successfully achieved percutaneous balloon dilation angioplasty for branch PAS in animal experiments and subsequent clinical studies (40–42). The initial low-pressure balloons (usually rated with burst pressures below 8 atm) were able to achieve technical success in 40–60% of patients (35, 38, 42–44). Later, the advent of relatively high-pressure balloons (36, 37, 45, 46) (rated with burst pressures >8 atm but no more than 15–22 atm) further increased the success rate to ~50–70%, making them the preferred choice to date. However, there are still a significant proportion of lesions that do not respond to simple BA (2).

Resistance to routine low- or high-pressure BA usually arises from one of the following conditions (2, 3, 44): (1) lesions that are too stiff to tear; (2) compliant lesions with some degree of retraction after angioplasty; and (3) stenoses due to compression of adjacent structures. In the first case, a higher-pressure balloon may theoretically be effective. A persistent waist prominent in the balloon during conventional low- or high-pressure balloon angioplasty is a reliable indicator of procedural failure. In such resistant lesions, the application of a larger balloon will further increase the incidence of complications rather than the success rate. Therefore, the UHP balloon is a good choice for this case. Initially developed for the treatment of stenotic hemodialysis fistulas (28), the UHP balloon catheters were rated with burst pressures as high as 30 atm. In practice, they can be inflated to pressures higher than 30 atm without rupture. In recent years, preliminary explorations have been carefully performed in infants and small children who were previously implanted with small stents. UHP BA was implicated to have the potential to treat resistant stenoses within or adjacent to pulmonary artery stents (32), to intentionally fracture the maximally dilated stents (29, 31), and to re-expand small diameter stents (30). These studies were mainly *in vitro* or animal experiments and clinical experiences in patients were still limited. In this study, we provided the first evidence for the safety and efficacy of UHP BA in the primary treatment of PAS in infants and children.

### 4.1. Efficacy of UHP balloon angioplasty

Our success rate (78.4%) was higher than historical data (35, 38, 42–44) with routine low-pressure (success rate: 29–50%) or high-pressure (success rate: 52–75%) balloons, indicating that higher pressure could be more effective to tear-resistant lesions. Given the learning curve for the first few cases, the success rate might be higher, approaching 80–90% according to our estimation.

The higher pressure imposed by the UHP balloons ensures a relatively higher rate of success in our series. The benefits of UHP balloons, however, were not only from the higher pressure but also from the ultra-non-compliance. The cross-layered ultrahigh molecular weight polyethylene material on the UHP balloon surface ensures the ultra-non-compliance of the balloons, which supported the stable position and shape of the balloon during angioplasty. In our experience, some resistant lesions could be successfully dilated by the UHP balloon, even with the same size and the same pressure (such as 8 atm) as the conventional balloon which failed. This could only be attributed to the higher non-compliance of UHP balloons.

The higher non-compliance might also help to decrease the risk of complications. During dilation of hard lesions, compliant balloons would have a prominent waist at the stenotic site and over-expansion at the adjacent normal vessel segment. In addition, the risk of injury to adjacent segments might elevate with increased balloon sizes. For non-compliant UHP balloons, we could succeed with a smaller size compared with a compliant balloon, thus potentially decreasing this risk.

### 4.2. Predictors of angioplasty success

Some predictors of success were previously summarized (2, 3), including vessel tears, size of balloons, and site of angioplasty (proximal or distal). Signs of vessel tears (therapeutic tears) were regarded as the most convincing evidence of successful dilation (36, 46), and the failure to tear the stenotic portion of the vessel (as revealed by a persistent waist in the balloon) was related to failed angioplasty (38). Previous histological (47) and imaging (34) studies suggested that the evidence of tear in the vessel intima and media was highly predictive of angioplasty success. This is consistent with our results. In our series, signs of therapeutic tears were strongly associated with technical success, with 100% of specificity and positive predictive values. There were also 21 cases in the success group that did not show signs of vessel tears. In these cases, vessel tears might be present but too small to be visualized from an angiographic view. Additional imaging technologies, such as intravascular ultrasound (34), as well as histological examinations, might provide extra evidence for effective tears. Therefore, there is no evidence of therapeutic tear observed during angiogram after the balloon dilation does not exclude angioplasty success.

The size of the balloon is another predictor of technical success. It is comprehensible that the larger the size of the balloon, the higher the possibility of a successful tear of vessel intima and media. However, too-large balloons have an increased risk of vessel rupture, leading to lethal complications such as mass airway bleeding. Previous experiences (3) recommended that the balloon/waist ratio to be around 2–3, but not exceeding twice the distal diameter. Our data supported this notion. The balloon/waist ratio was identified as an independent predictor of angioplasty success, and a ratio of 2.57 was best for the differentiation of success from failure. Our average ratio in the success group was 3.9, which was higher than the previous recommendations of 2–3, while the average ratio in the failure group was in the recommended range. Despite a higher ratio in our cohort, no peri-procedural death occurred.

Since only one angioplasty was performed at the distal site (right upper branch pulmonary artery) in our cohort, it is not feasible to analyze if UHP BA could be effective for distal lesions.

### 4.3. Safety of UHP balloon angioplasty

Adverse events were not uncommon in our group. Pulmonary artery injuries occurred in 30% of the total angioplasties. This is consistent with historical data (2, 25). Most of them, nevertheless, were signs of therapeutic tears, such as pulmonary artery vessel tears, vessel extravasations, and vessel intimal flaps. During follow-up, these signs were in a steady state and did not generate any adverse effects. Pulmonary artery aneurysms were relatively advanced adverse events and were present after two procedures. Both cases did not exaggerate during the follow-up to date. However, close monitoring is needed to detect in time the potential progression in the future. Pulmonary artery aneurysms, as well as signs of vessel tears, remained stable in the follow-up CTs or angiographies, indicating that effective vessel tear by UHP BA was not accompanied by subsequent spontaneous healing of the three-layered vessel structure. This is consistent with previous reports and confirmed with direct visualization of dilation sites in several patients who received secondary surgical repair. Severe adverse events in our series were rare. Pulmonary hemorrhage occurred in three cases. In addition, these three cases were all managed with conservative treatment, including mechanical ventilation with positive end-expiratory airway pressure. The amounts of bleeding were not large. All cases of hemorrhage terminated within 53 h followed by successful evacuation of ventilatory support. No adverse events related to heart rhythms or allergic reactions were seen in our group. Strikingly, the commonly reported complication of pulmonary edema was absent in our series. This might be attributed to our strategies with vigorous ventilatory support in CICU after angioplasty.

### 4.4. UHP balloon does not decrease the risk of restenosis

Restenosis occurred in approximately a quarter of cases in our group during a median follow-up period of 10.4 months (0.1–21.0 months). This is comparable to historical data with conventional low- or high-pressure balloons (rate of restenosis 10–44%) (35–38, 45, 46), which indicated that UHP balloons could not further decrease the incidence of restenosis. The exact mechanism of restenosis has not been fully elucidated. However, there is an association with angioplasty-induced vascular tears. Successful tearing of the intima and media is associated with successful dilation of the stenosis, and restenosis has been linked to a natural proliferative response to the vascular injury induced by BA. This response leads to deposition in the vascular matrix and ultimately restenosis. Some of the key factors involved in the response include the degree of induced injury, intima or media dissections, inherent elastic retraction of the vessel wall, and thrombus formation.

Predictive factors for restenosis have been investigated in previous studies (20, 46, 48), but the only risk factor understood to be associated with restenosis is the patient's weight gain during follow-up. Our data additionally indicated that a larger balloon/waist ratio was correlated with a greater risk of restenosis, which is not unexpected given that a larger ratio predicted a higher success rate. One of the explanations is that resistant lesions might be more likely to have a proliferative response to vascular injury. Larger balloons had a higher chance of tearing up resistant lesions than smaller balloons. In these resistant patients who succeeded with larger balloons, the proliferative response to vessel tears promoted restenosis during follow-up.

Stenting of the pulmonary artery was reported to have a lower rate of restenosis compared to balloon angioplasty (24). However, the majority of patients with PAS are infants and young children with the demand for somatic growth in the following years, which precludes stenting from being the first choice in this population. Restenosis could be treated with second balloon angioplasty or stenting. In recent years, UHP balloon angioplasty was also reported for stent re-dilation. They could be effective to open small stents and even cause intentional stent fractures to achieve the growing potential for pulmonary arteries. This pattern of serial intervention with repeated balloon angioplasty and stenting might be a good choice for refractory cases.

Taken together, the safety and efficacy of UHP balloons for primary angioplasty of PAS in infants and children had been demonstrated in this study. The angioplasty success rate was higher than historical data with conventional balloons, with a low incidence of severe adverse events. The predictors of success were a larger balloon/waist ratio and the presence of therapeutic tears. The occurrence of restenosis during follow-up, however, remained a problem that needed re-intervention with repeated balloon angioplasty or stenting. In the end, our data suggested that UHP balloon angioplasty should be considered for infants and young children with PAS as the procedure in priority.

### 4.5. Limitations

The present study had limitations. First, the case number of the study was relatively small. We need to expand the case number in the future to confirm the findings of this study. Second, the follow-up period was not long enough. We are unsure if the hemodynamic improvements in this study could last for a long period of time. It also remains to be elucidated if the restenosis would increase over time or become stable with time elapsing. Third, this is a retrospective clinical summary without controls. Comparisons were made with historical data. The safety and efficacy of UHP balloon angioplasty need further verification with a larger number of cases and better controls in the future.

## 5. Conclusion

The UHP BA is safe and effective for the primary treatment of PAS in infants and children with CHD. The success rate is high with a low incidence of severe complications. The predictors of success are a larger balloon/waist ratio and the presence of therapeutic tears. The occurrence of restenosis during follow-up, however, remains a problem. A larger number of cases and longer periods of follow-up are needed for further study.

## Data availability statement

The original contributions presented in the study are included in the article/supplementary material, further inquiries can be directed to the corresponding authors.

## Ethics statement

The studies involving human participants were reviewed and approved by Guangzhou Women and Children's Medical Center. Written informed consent to participate in this study was provided by the participants' legal guardian/next of kin. Written informed consent was obtained from the individual(s), and minor(s)' legal guardian/next of kin, for the publication of any potentially identifiable images or data included in this article.

## Author contributions

SX: conceptualization, data curation, investigation, and writing—review and editing. JL, ZW, and FL: data curation and investigation. LM: formal analysis and investigation. YC: data curation and modification. TL, XL, and SL: ultrasound diagnosis and evaluation. LS, LH, and XM: data curation. YL: image analysis. XC: conceptualization, investigation, and supervision. XZ: writing—review and editing, conceptualization, investigation, and supervision. All authors contributed to the article and approved the submitted version.
